# Supplementary material for: Hypokalemia is frequent and has prognostic implications in stable patients attending the emergency department
Source: PLoS One. 2020 Aug 4;15(8):e0236934. doi: 10.1371/journal.pone.0236934 (PMC7402484; doi:10.1371/journal.pone.0236934)
Supplement: S1 Table — eGFR, estimated glomerular filtration rate based on the CKD-EPI formula; Hb, hemoglobin. All variables with a p-value of less than 0.1 in Table 1 were introduced into the model. A backward selection process was conducted with 500x sampling bootstrap method. Variables were categorized to attain log-linearity. Hypokalemia defined as K+ <3.5 mmol/L (N = 148). Normokalemia defined as K+ 3.5–5.0 mmol/L (N = 1021). Hyperkalemia defined as K+ >5.0 mmol/L (N = 73). (DOCX) [file pone.0236934.s001.docx]

| Variable | Hypokalemia  OR (95%CI) | p-value | Normokalemia  (Reference) | Hyperkalemia  OR (95%CI) | p-value |
| --- | --- | --- | --- | --- | --- |
| Female gender |  |  | - | 0.47 (0.27-0.81) | 0.007 |
| eGFR <60 ml/min/1.73m^2^ |  |  | - | 4.16 (2.30-7.51) | <0.001 |
| Thiazide diuretics | 2.32 (1.28-4.17) | 0.005 | - | - | - |
| Hb <12 g/dL | - | - | - | 2.66 (1.52-4.62) | 0.001 |
| ACEi/ARBs | - | - | - | 1.89 (1.03-3.45) | 0.040 |
