# Supplementary material for: Hypokalemia is frequent and has prognostic implications in stable patients attending the emergency department
Source: PLoS One. 2020 Aug 4;15(8):e0236934. doi: 10.1371/journal.pone.0236934 (PMC7402484; doi:10.1371/journal.pone.0236934)
Supplement: Attachment — Submitted filename: Reviewers comments review 14 5.docx [file pone.0236934.s002.docx]

**Hypokalemia is frequent and has prognostic implications in stable patients attending the emergency department**

# **Answers to reviewers**

**Journal Requirements**

**1. Please ensure that your manuscript meets PLOS ONE's style requirements, including those for file naming. The PLOS ONE style templates can be found at**

**<http://www.journals.plos.org/plosone/s/file?id=wjVg/PLOSOne_formatting_sample_main_body.pdf>**

*Response:*

*Done*

**and** **<http://www.journals.plos.org/plosone/s/file?id=ba62/PLOSOne_formatting_sample_title_authors_affiliations.pdf>**

*Response:*

*Done*

**2. Please include additional information regarding the survey or questionnaire used in the study and ensure that you have provided sufficient details that others could replicate the analyses. For instance, if you developed a questionnaire as part of this study and it is not under a copyright more restrictive than CC-BY, please include a copy, in both the original language and English, as Supporting Information.**

*Response:*

*Thank you. The questionnaire has been previously published.*

*A copyright nevertheless prevents us from publishing it in our article. but we have now added the citation* ***and details about this questionnaire Lines 120-121 in the version with tracked changes and Lines 101-102 in the new version:***

***“This questionnaire was developed and validated by Roulet et al in 2008.”***

**3. In your Methods section, please provide additional information about the participant recruitment method and the demographic details of your participants.**

**Please ensure you have provided sufficient details to replicate the analyses such as:**

**a) a description of how participants were recruited, and b) descriptions of where participants were recruited and the names of the 11 French academic hospitals**.

*Response:*

*Thank you for that remark. The ADES-ED Cohort population was recruited from the emergency departments of 11 French hospitals. The participating centers were: CHU of Clermont-Ferrand, CHU of Caen, CHU of Toulouse, CHU of Nantes, CHU of Paris-Hôtel-Dieu Hospital, CHU of Rennes, CHU of Paris-Saint Antoine Hospital, CHU of Paris-Mondor Hospital, CHU of Grenoble, CHU of Paris-Cochin Hospital, and CHU of Angers (lines 87-90* ***in the version with tracked changes and lines 72-75 in the new version****).*

*All subjects 18 years of age and older who were likely to be admitted to a UHC emergency department during the study period, were recruited. The exclusion criteria were: deliberate drug intoxication; patient refusal to participate in the study; and patient's inability to give consent.*

*The study population was identified by a pair of students. We have added a section dedicated of recruitment method to the lines 91-96* ***in the version with tracked changes and lines77-86 in the new version:***

*“Recruitment method in ADES-ED cohort*

*The source population was all subjects 18 years of age and older who were likely to be admitted to a participating University Hospital emergency department during the study period.*

*The study population was identified by a pair of students (hospital medical students and fifth year hospital-university pharmacy students) trained in patient selection and data collection, with the help of the Nurse Organizer of the Reception Centre.*

*And lines 101-107* ***in the version with tracked changes and line 87-93 in the new version****:*

*“Criteria for inclusion:*

*All adult patients 18 years of age and older admitted to one of the 11 investigative centers during one of the collection periods.*

*Exclusion criteria:*

*- deliberate drug intoxication;*

*- patient refusal to participate in the study;*

*- patient's inability to give consent. “*

**4. We note that you have indicated that data from this study are available upon request. PLOS only allows data to be available upon request if there are legal or ethical restrictions on sharing data publicly. For information on unacceptable data access restrictions, please see [http://journals.plos.org/plosone/s/data-availability#loc-unacceptable-data-access-restrictions](http://journals.plos.org/plosone/s/data-availability#loc-unacceptable-data-access-restrictions).**

**In your revised cover letter, please address the following prompts:**

**a) If there are ethical or legal restrictions on sharing a de-identified data set, please explain them in detail (e.g., data contain potentially identifying or sensitive patient information) and who has imposed them (e.g., an ethics committee). Please also provide contact information for a data access committee, ethics committee, or other institutional body to which data requests may be sent.**

**b) If there are no restrictions, please upload the minimal anonymized data set necessary to replicate your study findings as either Supporting Information files or to a stable, public repository and provide us with the relevant URLs, DOIs, or accession numbers. Please see** [**http://www.bmj.com/content/340/bmj.c181.long**](http://www.bmj.com/content/340/bmj.c181.long) **for guidelines on how to de-identify and prepare clinical data for publication. For a list of acceptable repositories, please see** [**http://journals.plos.org/plosone/s/data-availability#loc-recommended-repositories**](http://journals.plos.org/plosone/s/data-availability#loc-recommended-repositories)**.**

**We will update your Data Availability statement on your behalf to reflect the information you provide.**

*Response:*

The dataset we used was a subset of the main *ADES-ED* study. Since there are ethical restrictions for data access per French Regulation (sensitive healthcare data), we got access to it via Mrs Trombert, acting as a deputy of the trial sponsor and of the ADES-ED trial steering committee. More broadly, requests for access require the agreement of the study investigators and the study sponsor studying the applicant's project. You can contact this committee by mail: Ms Béatrice Trombert-Paviot. ([Beatrice.TROMBERT-PAVIOT@chu-st-etienne.fr](mailto:Beatrice.TROMBERT-PAVIOT@chu-st-etienne.fr) ; CHU of Saint Etienne, France) or Pr Patrice Queneau ([pat-queneau@orange.fr](mailto:pat-queneau@orange.fr), Saint Etienne university, France).

5. Thank you for stating the following in the Financial Disclosure section:

'The National Medicine Academy approved the study design and provided funding. The French Society for Clinical Pharmacy (SFPC) approved the protocol, supported the enrolment and training of pharmacy students and provided funding. The French Association of Pharmaceutical Manufacturers for a responsible self-medication (AFIPA) approved the protocol and the conduct of the study. Sanofi Aventis approved the protocol and the conduct of the study. None of these funding sources intervened in the collection, management, analysis or interpretation of the data; nor in the preparation, review or approval of the manuscript.'

We note that you received funding from commercial sources: AFIPA and Sanofi Aventis

*Response****:***

*Our apologies for the confusion; the above statement actually was related to the main study, for which the initial authors also stated that “All authors have declared no potential conflicts of interest regarding this study.” In this post-hoc analysis, there is no funding, as clarified in the cover letter.*

a. Please provide an amended Competing Interests Statement that explicitly states this commercial funder, along with any other relevant declarations<<http://www.plosone.org/static/competing.action>> relating to employment, consultancy, patents, products in development, marketed products, etc.

Within this Competing Interests Statement, please confirm that this does not alter your adherence to all PLOS ONE policies on sharing data and materials by including the following statement: "This does not alter our adherence to PLOS ONE policies on sharing data and materials.” (as detailed online in our guide for authors <http://journals.plos.org/plosone/s/competing-interests>). If there are restrictions on sharing of data and/or materials, please state these. Please note that we cannot proceed with consideration of your article until this information has been declared.

*Response:*

*As explained above, there is no funding source for this post-hoc analysis. This is now clarified in the cover letter.*

b. Please include your amended Competing Interests Statement within your cover letter. We will change the online submission form on your behalf.

Please know it is PLOS ONE policy for corresponding authors to declare, on behalf of all authors, all potential competing interests for the purposes of transparency. PLOS defines a competing interest as anything that interferes with, or could reasonably be perceived as interfering with, the full and objective presentation, peer review, editorial decision-making, or publication of research or non-research articles submitted to one of the journals. Competing interests can be financial or non-financial, professional, or personal. Competing interests can arise in relationship to an organization or another person. Please follow this link to our website for more details on competing interests: <http://journals.plos.org/plosone/s/competing-interests>

*Response:*

*Done.*

6. PLOS requires an ORCID iD for the corresponding author in Editorial Manager on papers submitted after December 6th, 2016. Please ensure that you have an ORCID iD and that it is validated in Editorial Manager. To do this, go to ‘Update my Information’ (in the upper left-hand corner of the main menu), and click on the Fetch/Validate link next to the ORCID field. This will take you to the ORCID site and allow you to create a new iD or authenticate a pre-existing iD in Editorial Manager. Please see the following video for instructions on linking an ORCID iD to your Editorial Manager account: <https://www.youtube.com/watch?v=_xcclfuvtxQ>

*Response:*

*Dr Rossignol ORCID number is <https://orcid.org/0000-0001-8009-3873>*

**Reviewers' comments**

**Reviewer #1**

**The following information needs to be included in the manuscript:**

1. **The inclusion criteria of the ADES-ED study. Without it, the study group is not clearly defined**.

*Response:*

*Thanks; we proceeded accordingly and inserted the following text in order to clarify the criteria of the ADES-ED study lines 101-107* ***in the version with tracked changes and lines 87-93 in the new version****:*

*“Criteria for inclusion:*

*All adult patients 18 years of age and older admitted to one of the 11 investigative centers during one of the collection periods.*

*Exclusion criteria:*

*- deliberate drug intoxication;*

*- patient refusal to participate in the study;*

*- patient's inability to give consent. “*

2. **In line 102, a standardized survey was used. How was the survey validated?**

*Response:*

*The questionnaire has been previously published (Roulet et al, reference 27), with a copyright preventing us from publishing it within the present paper.*

*This Questionnaire to document self-medication was developed between January and September 2008 by Roulet et al; during the same period, spontaneous report of self-medication use was collected (“reference period”). The questionnaire was tested and reﬁned between October and December 2008 (“test period”). Finally, the QSMB was routinely used between January and December 2009 (“routine period”). After verifying that the patients interviewed during the reference and routine periods were not signiﬁcantly different with regard to baseline characteristics, Roulet et al assessed the utility of questionnaire by comparing the self-medication rates measured during these two periods.*

*We have now quoted this paper* ***lines 119-120 in the version with tracked changes and lines101-102 in the new version:***

***“This questionnaire was developed and validated by Roulet et al (27).”***

3. **Line 116 mentions the PARADISE cohort. A detailed description of the cohort is needed. Why was this group chosen?**

*Response:*

*Thank you for this remark; we now more accurately describe the PARADISE cohort. This cohort "Pathway of dyspneic patients in Emergency”, consisting of including all patients over one year aged 18 years or older admitted to the ED of the Nancy University Hospital (France) for dyspnea. This cohort included a total of 1,589 patients, including 1318 with a potassium measurement.*

*This cohort was chosen for several reasons:*

*- It is a French population of patients presenting in the emergency department.*

*- Our team made up this cohort. So we had easy access to the data.*

*We have added the sentence to lines 247-250* ***in the version with tracked changes and lines 222-225 in the new version****:*

*“For external validity purposes, we compared our prevalence results with another ED French cohort - the PARADISE cohort "Pathway of dyspneic patients in Emergency”, consisting of including all patients over one year aged 18 years or older admitted to the ED of the Nancy University Hospital (France) for dyspnea., as previously reported by our group (29).”*

4. **Lines 106-107: describe why there are two different measures for hypokalemia being used for the study**.

*Response:*

*Thank you. These cut-offs were chosen to have a better insight on the shape of the associations, as most authors define a potassium level <4mmol/L as hypokalemia (reference 29), but potassium levels <3.5mmol/L may have a stronger prognostic association, and were selected by other groups e.g. recently by Cooper et al Eur J Heart Fail. 2020 (reference 30).*

*Therefore, we wanted to assess both.*

*We have added the sentence to lines 126-129* ***in the version with tracked changes and lines 109-112 in the new version****:*

*“We chose to use two thresholds because most authors define a potassium level <4mmol/L as hypokalemia [29], but potassium levels <3.5mmol/L may have a stronger prognostic association and were considered by others [30].”*

*We now also state that, line 130* ***in the version with tracked changes and lines 112-113 in the new version*** *that:*

*“normal potassium range was considered when potassium levels were between 4 and 5mmol/L or 3.5 to 5mmol/L depending on the threshold considered”*

5. **My previous comment also relates to the two tables (3 & 4) looking at outcomes between a potassium less than 4 and 3.5. It is not clear to me why you need two tables**.

*Response:*

*We have made two tables since there are actually two different analyses. Indeed, the association of K+<3.5 with outcomes is stronger, therefore, we would like to maintain it for the reader*.

6. **In the discussion section, you need to address the fact that your population included only those who had blood drawn. This inherently selects for those who are likely sicker than those who did not have blood drawn**.

*Response:*

*Great point, thank you. We have added this sentence in limitation’s section lines 293-294* ***in the version with tracked changes and lines 264-265 in the new version****:*

*“Lastly, our population only included people who have had a blood test with electrolytes. This fact selects patients who are probably sicker than those who did not have blood tests.”*

**Reviewer #2**

**This study by Ferreira et al. investigated the prevalence of dyskalemia in ED of multiple medical centers/hospitals across the France. This was done in a prospective and cross-sectional manner. It is interestingly showed that hypokalemia occurs most often in ED patients while hyperkalemia is rare. Though hypokalemia sounds like “low-risk” but is associated worse outcomes in terms of hospitalization and death. While this is very interesting, there are a couple of issues to be addressed:**

1. **First of all, according to Table 1, there is more frequent hospitalization (including in ward and critical care) of patients with hyperkalemia than of patients with hypokalemia or normokalemia. This agrees with more treatments with cardiovascular drugs such as ACEi, CCB, and diuretics, suggesting these patients had CVD more frequently (e.g., hypertension). Please explain this is contradicting with Table 4. In Table 4, the crude OR was 1.51 for the association between hyperkalemia and outcomes but adjusted OR showed an OR<1.0 (0.61). Why is that and what does this mean?**

*Response:*

*This point is well taken. In the Table 4, we adjusted on age, gender, estimated glomerular filtration rate, hemoglobin and Thiazide. Indeed, patients with K>5 were older, had lower eGFR and lower hemoglobin, all of which are associated with higher risk for death.*

*When adjusting on those factors, the increase in the risk of event observed with K>5 is reversed to a rather preventive association (lower than 1). This is because of the major confounding effect of age, eGFR and hemoglobin.*

*In the revised version of the manuscript we added this sentence to provide this important message to the reader, in the methods section lines 149-151* ***in the version with tracked changes and lines 129-131 in the new version****:*

*« We adjusted on age, gender, estimated glomerular filtration rate, hemoglobin and Thiazide: patients with K>5 were indeed older, had lower eGFR and lower hemoglobin (Table 1), all of which being associated with higher risk for death. »*

2. **Why in Table 3 and Table 4, K+>5.0 had different OR and p values for the outcome of hospitalization or death?**

*Response:*

*The association of K>5 with hospitalization/death is changing from one table to the other because of a different reference category. In the Table 4, the reference group (3.5-5) is larger, and at lower risk for event than the reference group (4-5). This directly increases the association of K>5 with outcome in Table 4 vs Table 3. We have now made this clearer in the manuscript by making clear what is the reference category in the methods section (stating now, line 129* ***in the version with tracked changes and line 112 in the new version*** *that “normal potassium range was considered when potassium levels were between 4 and 5mmol/L or 3.5 to 5mmol/L depending on the threshold considered”) and in the respective tables.*

*Please see the revised version of the manuscript.*

3. **The comparison between your study with the French cohort of 1318 patients should be removed from the abstract and the results part. It is appropriate to keep that in the discussion.**

*Response:*

*We proceeded accordingly and removed this part from the abstract, and these points are detailed in the discussion section. Please see the revised version of the manuscript.*

**4. What does MRAs stand for in Table 1? Please make sure you have spelled out all abbreviations in the text and notes for tables**.

*Response:*

*Thank you. MRAs stands for mineralocorticoid receptor antagonists. We have checked all abbreviations and added them to the respective legends of the tables. Please see the revised version of the manuscript.*
